# Factors affecting the quality of life for patients with end-stage renal disease on dialysis in KwaZulu-Natal province, South Africa: A descriptive survey

**DOI:** 10.4102/hsag.v27i0.1932

**Published:** 2022-12-07

**Authors:** Pretty N. Mbeje

**Affiliations:** 1 School of Nursing and Public Health, Faculty of Health Sciences, University of KwaZulu-Natal, Durban, South Africa

**Keywords:** quality of life, end-stage renal disease, haemodialysis, peritoneal dialysis, South Africa

## Abstract

**Background:**

End-stage renal disease (ESRD) is a world-wide public health problem that requires renal replacement therapy in the form of dialysis. Although dialysis prolongs the patients’ lifespan, it is not necessarily associated with an improved quality of life (QoL).

**Aim:**

To determine the factors affecting the QoL of patients with ESRD on dialysis.

**Setting:**

The study was conducted in three public sector hospitals in the province of KwaZulu-Natal.

**Methods:**

A convenience sample of 316 participants was used. The World Health Organization – QoL Biomedical Research and Education Foundation (WHOQOL-BREF) instrument was used and adapted to include economic factors. Data were analysed using descriptive and inferential statistics.

**Results:**

Majority had poor QoL through economic (98.1%), psychological (94.6%), physical (70.3%), and social factors (55.1%). Factors affecting their QoL were influenced by the type of dialysis, and statistical significances were noted in psychological and social factors (*p* < 0.0001), with those on peritoneal dialysis being affected the most. Overall, majority of patients (91.7%, *n* = 288) had a score of less than 50, which indicated poor QoL.

**Conclusion:**

Poor QoL is associated with increased risk of mortality and hospitalisation in patients with ESRD and is mainly influenced by a broad range of dimensions of life.

**Contribution:**

In addressing challenges encountered by patients, study findings may influence the policymakers to intervene in view of all the dimensions of QoL, to prolong life.

## Background

End-stage renal disease (ESRD) is a global health challenge from which, according to the Global Burden of Disease Study in 2017, 1.2 million people have died, and all-age mortality rate has increased to 41.5% between 1990 and 2017 (Bikbov et al. 2020). Globally, the prevalence is estimated to be 0.07%, or approximately 5.3 million people in 2017, with other estimates ranging as high as 9.7 million (Himmelfarb et al. 2020). In 2013, the World Health Organization (WHO) provided treatment guidelines that included adherence to renal replacement therapy, taking medication, following a strict renal diet, and adjustments to lifestyle that included physical, social, psychological, and economic dimensions (Luyckx, Tonelli & Stanifer 2018).

Haemodialysis and peritoneal dialysis are the only dialysis treatment options for patients with ESRD, both placing a considerable physical, psychological, economic, and social strain on the patients, resulting in a reduced quality of life (QoL) (Hackett & Jardine 2017). Haemodialysis is an extracorporeal blood cleansing technique delivered via an inserted haemodialysis catheter to remove metabolic wastes for patients with renal failure (Ronco & Clark 2018). On the other hand, peritoneal dialysis involves infusing dialysate fluid into the peritoneal cavity via the inserted Tenckhoff catheter, where it dwells for a period of 4 h – 5 h before it is drained out (Mogotlane 2017). Furthermore, peritoneal dialysis fluid is costly, yet it is used in patients with better residual functioning of the kidney. Moreover, peritoneal dialysis is much better when compared to haemodialysis, which raises the need to be investigated because the fluids are locally produced (Moosa et al. 2016). In South Africa, although peritoneal dialysis is more expensive than haemodialysis, it is the preferred option, as it allows patients to be trained to perform it at home, given the unavailability of health care resources (Makhele et al. 2019).

Patients with ESRD and on dialysis are dependent on their families, friends, and important others for their survival (Sousa et al. 2019). They often suffer from depression caused by loneliness experienced during the course of treatment (Chen, Wang & Lang 2016). Most of the patients struggle financially as a result of low or no income and the cost of travelling to facilities that provide renal treatment. In South Africa, patients from poor socio-economic backgrounds and remote and rural areas are mostly affected financially, as they have to travel long distances to access institutions that render renal therapy, which are concentrated in urban areas (Li et al. 2017). End-stage renal disease results in poor QoL for the affected patients, particularly those on dialysis (Varghese 2018). Fradelos (2020) defines QoL as:

[*T*]he situation in which human life is characterized as good on the basis of two components: (1) the patient’s ability to perform activities that require physical, mental and social well-being and (2) to satisfy the individual with regard to his or her functionality and disease control. (p. 2)

The general health of patients with chronic kidney disease is affected by the stage of the disease, with ESRD being the last stage of this debilitating illness (Gilardino et al. 2018). Obligation to self-care, medication administration, fluids, and dietary management result in psychological strain leading to poor motivation, nonadherence, and deteriorating QoL (Lim et al. 2016).

In sub-Saharan Africa, there is a high prevalence rate of 13.9% of the population suffering from comorbid diseases like hypertension and diabetes. Hence, the health care providers tend to focus more on management of these diseases without considering their complications, which then leads to delayed diagnoses of renal failure (Moosa et al. 2016). Tannor et al. (2017) cited that Africa has 97% of patients in dialysis of which South Africa has the highest prevalence of 8% – 16%. Although unfavourable home conditions have been cited as hindering access to care, there has been comparatively little documentation of the challenges encountered by patients with ESRD on dialysis (Tannor et al. 2017).

Little has been documented regarding factors affecting the QoL of patients with ESRD on dialysis (Mbeje & Mtshali 2019). Quality of life should be measured frequently and appropriate interventions applied; however, this is not frequently done (Chen et al. 2016). Assessing and treating symptoms positively influences the patient outcomes (Chen et al. 2016). This study has therefore explored the factors affecting the QoL of patients with ESRD in the public sector hospitals of KwaZulu-Natal that render renal services to the greater part of the province. As ESRD is a global challenge, this study aims at determining the factors affecting the QoL of patients with this condition, who are on haemodialysis or peritoneal dialysis within the context of KwaZulu-Natal, South Africa. The results will enable the researchers to develop a framework which could be used by the policymakers to address the challenges encountered by patients with ESRD on dialysis.

## Methodology

### Study design

A cross-sectional descriptive quantitative design was used to determine the factors affecting the QoL of patients with ESRD on haemodialysis or peritoneal dialysis.

### Study setting

The study was conducted in three public sector hospitals in eThekwini municipality, and one in Umgungundlovu district in KwaZulu-Natal. These are the public institutions which provide renal services to the greater part of the province of KwaZulu-Natal. One institution in Ethekwini municipality is at a tertiary level and provides all renal services for the entire province. The other two institutions in eThekwini municipality provide only haemodialysis services. The institution in Umgungundlovu district provides haemodialysis services and has a renal ward for inpatient management.

### Population and sampling

During data collection in the selected hospitals, there was a total population of 525 patients with ESRD on dialysis. The following parameters were used to determine the sample size: Effect size = 0.18; Type I (α) error of 0.05 (i.e. the probability of falsely rejecting the null hypothesis is 5%); Type 2 (β) error (i.e. false negative); statistical power is 1 – β which is 1–0.2 = 0.8 (80%). On the basis of the above statistical parameters, a sample size of 316 was found to be realistic.

A total of 316 patients with ESRD were selected to participate from four facilities, with 58.9% (*n* = 186) being from Hospital A (tertiary), 17.1% (*n* = 54) from Hospital B (tertiary), 15.5% (*n* = 49) from Hospital C (regional), and 8.5% (*n* = 27) from Hospital D (regional). Convenience sampling was used, as these are the only public sector institutions in KwaZulu-Natal province that offer dialysis services to patients with ESRD. The researcher used the available patients that met the research criteria and were either on haemodialysis or peritoneal dialysis, as they attended the outpatient renal clinics (for peritoneal dialysis follow-up appointments) or were admitted to hospital for haemodialysis sessions. Convenience sampling was employed because of the complicated nature of ESRD, where it is not guaranteed that the patients will come to renal facilities for their next visits as anything may adversely happen to them.

#### Inclusion criteria

All participants who were fully conscious, 18 years old or above and diagnosed with ESRD, on any form of dialysis, from KwaZulu-Natal, and in the renal units of selected hospitals were included in the study. All the selected participants agreed to participate and were available at the time of study.

#### Exclusion criteria

Patients in the renal unit but from outside the province of KwaZulu-Natal, those who were not diagnosed with ESRD, and those with ESRD but not on any form of dialysis were excluded from the study. Patients who already had kidney transplants were excluded, as their QoL is different from those who are on dialysis. Moreover, the patients who were available but not feeling well during the time of the study and those who were younger than 18 years of age were excluded.

### Research instrument used

Data were collected using a structured questionnaire adapted from the World Health Organization Quality of Life Biomedical Research and Education Foundation (WHOQOL-BREF) to determine the factors that affect the QoL of patients with ESRD (Kim 2021). A major part of the environmental factors which were part of the WHOQOL-BREF instrument were excluded. The researcher only considered the economic factors which were within the environmental component as they were relevant to the context of KwaZulu-Natal province. The questions used in the instrument were guided by the aim of the study. The research instrument had the sociodemographic data and 35 items grouped into five factors: Physical (12 items), psychological (5 items), social (6 items), economic (7 items), and general health domains (5 items). The scoring system used for raw data was 0–5.

### Validity and reliability of the research instrument

The researcher used the WHOQOL-BREF instrument, which is ideal for QoL research studies and was approved by the WHO, which ensured good internal consistency. The face validity of the instrument was shown through the instrument, which was structured in demographics, physical, psychological, social, economic and general health-related conditions. The researcher used in-depth literature to modify the instrument according to the study context. The instrument was sent to the supervisor and other experts in the field to check the content. The Biomedical Research Ethics Committee (BREC) also reviewed the adapted instrument and assessed whether it addresses the research aim. The concepts were clearly presented and easily interpreted with intended factors. The WHOQOL-BREF instrument was validated with a reported Cronbach’s alpha coefficient of 0.89.

### Data collection

Data were collected from the four identified public institutions which offer renal replacement therapy in the province of KwaZulu-Natal for a period of 6 months, from October 2017 to March 2018. Prior to data collection and before the participants signed the informed consent, the researcher briefly explained the study and its purpose to them. The actual distribution and collection of research questionnaires was done by the unit managers. In responding to the questionnaire, the participants selected their responses from the options given in the tool, which took 15 min – 20 min to complete. The response rate was 100%.

### Data analysis

The data were analysed using the International Business Machines (IBM) Statistical Package for the Social Sciences (SPSS) software version 24. The raw data for each factor were transformed prior to data analysis and put on a scale ranging from 0 to 100, in which a score of below 50 indicated poor QoL. The overall scores for each of the five factors were calculated and their descriptive statistics displayed using a box plot. The findings were presented using frequencies, percentages, minimum and maximum scores, mean and standard deviations, medians, and quartiles. The Mann–Whitney *U* test was used to establish the association of QoL and the type of dialysis, and the Pearson correlation coefficient was used to explore the relationships between the five factors affecting QoL with the level of significance being *p* < 0.05.

### Ethical consideration

Ethical principles were adhered to as the study commenced only after obtaining ethical clearance from the Biomedical Research Ethics Committee (BREC) of the University of KwaZulu-Natal (ref. no. BE506/17), which enabled the researcher to obtain the necessary permission from the management of the four public health institutions. Data collection commenced after obtaining informed consent from the participants, who also had the right to withdraw from participation at any time. Patients’ rights were respected, as those who were not feeling well were excluded from the study to prevent them from discomfort and harm. Privacy and confidentiality were maintained, as data could not be traced back to the study participants as in line with POPIA Act 4 of 2013 as amended (Staunton & De Stadler 2019).

## Results

A total of 316 patients with ESRD participated in this study, of whom 53.8% (*n* = 170) were on haemodialysis and 46.2% (*n* = 146) were on peritoneal dialysis. The demographic characteristics (Table 1) indicate hospitals, age group, gender, level of education, area of residence, and employment status.

**TABLE 1:** Demographic characteristics.

Variables	Frequencies (*n*)	Percentage of patients (%)
**Hospital**		
Hospital A	186	58.9
Hospital B	54	17.1
Hospital C	49	15.5
Hospital D	27	8.5
**Age group (years)**		
< 40	120	38.0
40 and above	196	62.0
**Gender**		
Female	152	48.1
Male	164	51.9
**Level of education**		
No education	7	2.2
Primary education	50	15.8
Secondary education	202	63.9
Tertiary education	57	18.0
**Residential area**		
Suburb	34	10.8
Rural	117	37.0
Location or township	165	52.2
**Employment status**		
Unemployed	245	77.5
Self-employed	37	11.7
Part-time employed	26	8.2
Full-time employed	8	2.5

The findings indicated that several factors affected the QoL of ESRD patients who were on dialysis. The results were grouped into five factors: Physical, psychological, economic, social, and general health. Overall scores for each of the five factors were calculated, with the higher scores indicating a better QoL.

### Physical factors

Twelve physical factors were explored in this study, with six factors having a mean score of less than 50, indicating poor QoL. The factors were as follows: Hypertension (6.96 ± 25.49), surgical operations (8.25 ± 27.56), losing weight after starting dialysis (15.18 ± 25.94), getting tired from the dialysis (14.71 ± 24.49), getting darker in complexion after starting dialysis (24.92 ± 3.24), and frequency of admission for dialysis (33.30 ± 15.43). Physical factors with a mean score of above 50 were diabetes (67.72 ± 46.82), infections (53.96 ± 49.92), special procedures (65.71 ± 47.54), uncontrolled hypertension (88.25 ± 32.24), uncontrolled diabetes (97.14 ± 16.68), and other conditions (57.46 ± 49.51).

**FIGURE 1:**
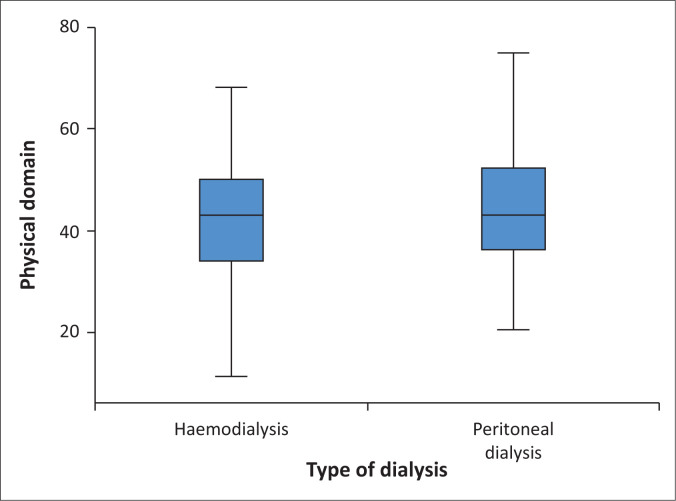
Impact of physical factors on quality of life of patients on dialysis.

In order to explore the impact of physical factors on QoL, 12 items were computed and an overall score calculated, with the minimum score being 11.36 and the maximum 75. The mean and standard deviation were 43.24 ± 11.66, and the median was 43.18. Quartile 1 was 36.36, Quartile 2 was 45.18, and Quartile 3 was 50. The results indicated that regarding the physical factors that affected the QoL, 70.3% (*n* = 222) had a poor QoL as they scored less than 50. Although slight differences were noted regarding the impact of physical factors on the QoL of the patients on haemodialysis or peritoneal dialysis, the Mann–Whitney *U* test indicated that those differences were not statistically significant (*U* = 11688.00, *p* = 0.260). Patients on peritoneal dialysis had a score of 44.25 ± 11.61, with a minimum of 20.45 and a maximum of 75, and those on haemodialysis scored 42.38 ± 11.63, with a minimum of 11.36 and a maximum of 68.18. In both types of dialysis, the median was 43.181 (Figure 1).

### Psychological factors

**FIGURE 2:**
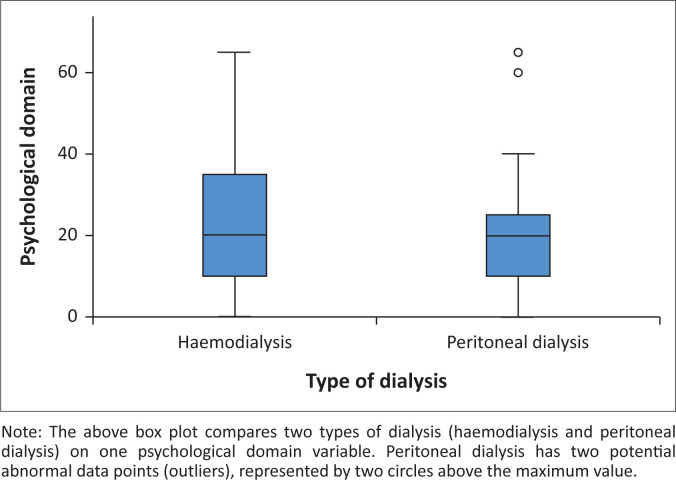
Impact of psychological factors on quality of life of patients with end-stage renal disease and on dialysis.

All the psychological factors explored in this study scored less than 50, indicating that they negatively affected the participants’ QoL. The factors were as follows: Experiencing a lack of self-confidence (7.59 ± 26.53), being in denial after being informed that both kidneys were affected (14.55 ± 25.15), feeling depressed after being informed about the need for dialysis (17.16 ± 25.19), feeling anxious about the family and their own future (17.80 ± 21.11), and having a lowered self-esteem caused by the presence of surgical scars or insertion of the dialysis catheters (46.99 ± 39.42) (Figure 2).

The overall score was calculated after computing the five psychological factors, with the minimum score being 0 and the maximum 80; the mean and standard deviation were 20.71 ± 14.83, and the median and mode were both 20. Quartile 1 was 10, Quartile 2 was 20, and Quartile 3 was 30. The QoL of the patients was negatively affected by the psychological factors, with the majority scoring less than 50. Overall, 94.6% (*n* = 299) had a poor QoL caused by psychological conditions.

The impact of psychological factors on those on peritoneal or haemodialysis was explored. Although patients who were on either type of dialysis had poor QoL, those on haemodialysis scored higher, with a median of 20, their mean and standard deviation being 23.26 ± 16.66, the scores ranging from 0 to 80. Those on peritoneal dialysis had a median of 20, a mean and standard deviation of 17.97 ± 12.76 and scores ranging from 0 to 70. The Mann–Whitney *U* test indicated that the differences between the types of dialysis were statistically significant (*U* = 10096.500, *p* = 0.004). Those findings indicated that patients on peritoneal dialysis had a more compromised QoL than those on haemodialysis.

### Economic factors

The participants’ QoL was severely affected by economic factors, with a number of variables scoring below 50. These factors were as follows: The impact of dialysis on their personal (1.26 ± 11.19) and family economy (1.89 ± 13.66), drastic changes to the family income (4.43 ± 14.23), financial constraints caused by clinic appointments and dialysis (5.06 ± 16.00), challenges finding transport from home to the hospital for appointments or dialysis (12.81 ± 29.51), loss of employment caused by having chronic ESRD (49.68 ± 5.07), and poor finances resulting in their missing the clinic appointments or dialysis (48.57 ± 34.18). Seven items were computed and the scores calculated to explore the overall impact of the psychological factors on QoL.

**FIGURE 3:**
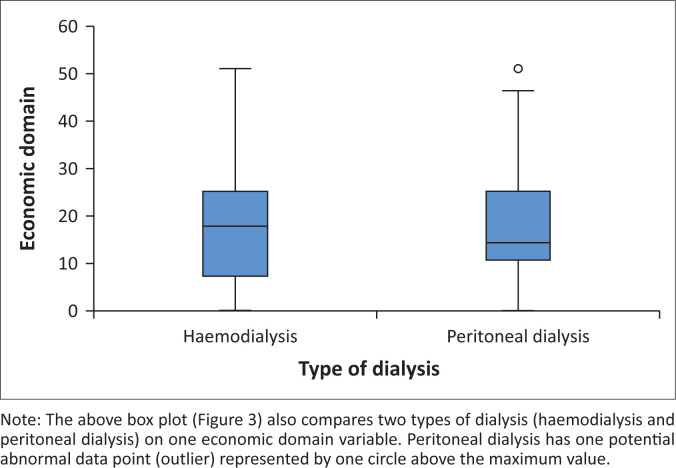
Impact of economic factors on the quality of life of patients with end-stage renal disease and on dialysis.

The minimum score was 0 and the maximum 51. The median was 17.85, the mode 3.57, the mean and standard deviation were 17.51 ± 11.95. Quartile 1 was 7.14, Quartile 2 was 17.85, and Quartile 3 was 25. Overall, 98.1% (*n* = 310) of patients had poor QoL caused by economic factors. Patients on peritoneal or haemodialysis were affected equally by economic factors; however, there were no statistically significant differences, as demonstrated by the Mann–Whitney *U* test (*U* = 12079.00, *p* = 0.0681). It was noted that the median score (17.85) was higher for patients on haemodialysis (the mean and standard deviation were 17.28 ± 12.11), than those on peritoneal dialysis, with the median being 14.28, and mean 17.78 ± 11.80 (Figure 3).

### Social factors

Various social factors negatively affected their QoL, as indicated by a mean score of less than 50. This was indicated by the following: Dialysis depriving them of engaging in various social activities (5.69 ± 17.57), their changed social roles caused by the effects of chronic ESRD (34.81 ± 29.45), and being a burden to the family (48.33 ± 45.72). Among other social factors affecting their QoL were the weekly frequency of haemodialysis (57.91 ± 43.10), or peritoneal dialysis (65.26 ± 37.56).

An overall score was calculated after computing the five items related to social factors affecting their QoL, with a minimum score of 16.67 and a maximum of 83.33. The mean and standard deviation were 42.24 ± 16.04, the median was 50 and the mode 58.33. The findings indicated that Quartile 1 was 16.67, Quartile 2 was 50, and Quartile 3 was 58.33. Overall, 55.1% (*n* = 174) scored less than 50, indicating a poor QoL caused by social factors, and 44.9% (*n* = 142) had good QoL as a result of social factors.

**FIGURE 4:**
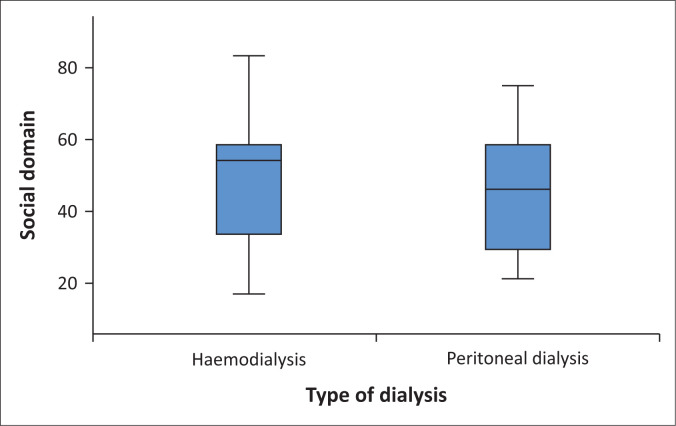
Impact of social factors on the quality of life of patients with end-stage renal disease and on dialysis.

The findings indicated higher social factor scores in patients on haemodialysis (mean and s.d. = 48.08 ± 16.27, median = 54.16, the minimum = 16.67 and the maximum = 83.33), than in those on peritoneal dialysis (mean and s.d. = 44.09 ± 15.55; the median = 45.83), with a minimum of 20.83 and a maximum of 75 (Figure 4). The Mann–Whitney *U* test indicated that the differences were statistically significant (*U* = 10636.000, *p* = 0.005).

### General health-related conditions

**FIGURE 5:**
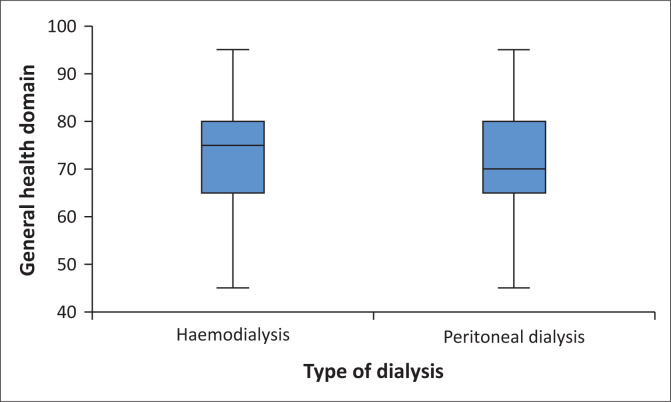
Impact of general health conditions on the quality of life of patients with end-stage renal disease and on dialysis.

The findings indicated that the domain of general health conditions affected their QoL the least compared to other domains. The state of health of patients compared to the previous 2 years (47.06 ± 37.18), indicates that general health did not affect their QoL (Figure 5). Other factors were as follows: ESRD being the cause of poor health (95.63 ± 14.06), the health status deteriorating at any time (93.41 ± 15.13), feeling that one’s health status was good (51.58 ± 36.31), and being prone to illnesses in general compared to other people (78.41 ± 28.25). The overall score was calculated to explore the impact of general health on the QoL of patients with ESRD, with the minimum being 45 and the maximum 95. The mean and the standard deviation were 73.20 ± 10.30, the median was 75 and the mode 80. It was observed that Quartile 1 was 65, Quartile 2 was 75, and Quartile 3 was 80. Overall, 95.9% (*n* = 303) of patients had an excellent QoL in relation to general health conditions.

In relation to the type of dialysis, the patients on haemodialysis had a median score higher (75) than those on peritoneal dialysis (70). Furthermore, the mean score for haemodialysis was 73.78 ± 11.06, with the score ranging from 45 to 95. For patients on peritoneal dialysis, the mean score was 72.53 ± 9.34, which ranged from 45 to 95. However, those differences were not statistically significant, as demonstrated by the Mann–Whitney *U* test (*U* = 11129.00, *p* = 0.129).

An overall score of QoL of the patients with ESRD was calculated after computing the 34 factors affecting the QoL. The responses ranged from 0 to 100, with the higher score indicating a good QoL. The minimum score was 23.77, the maximum was 59, the mean was 40.22, the standard deviation was 6.74, the mode was 33.23 and the median was 39.98. It was noted that the Quartile 1 was 35.70, Quartile 2 was 39.98, and Quartile 3 was 44.29.

**FIGURE 6:**
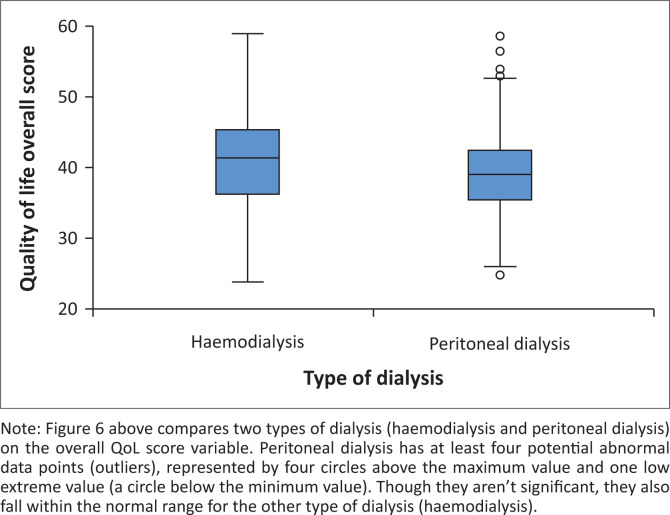
Overall quality of life of patients with end-stage renal disease and on dialysis.

Overall, the majority of patients with ESRD (91.7%, *n* = 288), had a score of less than 50, which indicated a poor QoL. However, statistically, differences were noted between the type of dialysis and the QoL. A lower mean score in peritoneal dialysis (39.37 ± 5.90) and median of 24.69 was observed. However, a higher mean score was shown in haemodialysis (40.95 ± 6.86) with a median of 23.77 (Figure 6).

The economic domain had the lowest scores indicating poor QoL (17.67 ± 12.48), followed by the psychological domain (20.82 ± 15.19), physical (43.24 ± 11.66), and social domains (46.24 ± 16.04). The general health domain scored high in the patients with ESRD (73.20 ± 1.30), with the Pearson correlation coefficient indicating a significant relationship between domains of QoL of patients with ESRD. Statistically significant positive correlations were reported between the physical and economic (*r* = 0.218, *p* < 0.001), physical and social (*r* = 0.168, *p* < 0.001), psychological and social (*r* = 0.177, *p* < 0.001), and economic and social domains (*r* = 0.188, *p* < 0.001). There was a significant negative correlation between the general health and physical (*r* = −0.149, *p* < 0.001), and general health and economic domains (*r* = −0.236, *p* < 0.001).

## Discussion

The study showed that 68% of the participants were above the age of 40 years. This is supported by McCullough et al. (2019), who projected an accelerated increase of patients with ESRD above 40 years by the year 2030. This study revealed that of the participants, 77.5% were unemployed and 37% lived in rural areas. Jardine et al. (2020) stated that South Africa, as a developing country, has a greater proportion of rural patients on ESRD, and in 2030, 70% of the population will be from low-income groups.

The reported factors affecting the QoL included physical, psychological, economic and social factors, as well as general health-related conditions. In this study, the majority of the patients with ESRD had a poor QoL caused by physical factors (70.3%, *n* = 222). Underlying diseases such as hypertension, diabetes, infections, and the impact of the invasive procedures used to treat ESRD contributed to their poor QoL. This association was also reported by Zaki, Alashwal and Ibrahim (2020), who recommended the provision of optimal treatment to ensure better outcomes and improved QoL for patients suffering from comorbidities. Additionally, patients with uncontrolled comorbid conditions are frequently hospitalised to stabilise their conditions, which leads to poor clinical outcomes and even mortality (Lim et al. 2016).

In this study, 94.6% (*n* = 299) had a poor QoL caused by psychological conditions such as depression, denial, anxiety, stress, and a lack of self-confidence. The findings in this study substantiate the literature, which indicated that both treatment modalities place an enormous psychological strain on patients, thereby resulting in lower QoL (Hackett & Jardine 2017). Emotional well-being in patients with ESRD on haemodialysis or peritoneal dialysis is affected by depression, which increases the possibility of developing symptoms of anxiety (Rajan & Subramanian 2016).

Additionally, Lim et al. (2016) reported that body image and physical limitations hinder patients from fulfilling their social roles, which contributes to lowered self-esteem. This is worsened by a lack of self-confidence, especially when their role as economic provider for the family changes suddenly (Lim et al. 2016). Similarly, Tannor et al. (2017) argued that patients on peritoneal dialysis experience low self-esteem and embarrassment caused by the Tenckhoff catheter which always hangs from their abdomen. This results in denial of the illness, which sometimes causes nonadherence to dialysis. This in turn leads to anxiety, frustration, withdrawal, and depression. As such, renal health care providers need to be vigilant of their patients’ needs and provide continuous guidance, support and education, empowerment, or affiliation (Lim et al. 2016).

Poor social support plays a major role in the deterioration of the QoL of patients with ESRD (Chen et al. 2016). The findings in this study revealed that 55.1% (*n* = 174) scored less than 50, indicating poor QoL caused by social factors. Patients with ESRD frequently use replacement therapy, such as haemodialysis or peritoneal dialysis, which hinders them from engaging in social activities, and leads to them feeling lonely, rejected, and burdensome to the family (Chen et al. 2016). The literature indicated that good family relationships are significantly associated with emotional comforts, and they greatly influence the QoL (Kukihara et al. 2020). In this study, it is statistically significant that a difference was noted in the social domain, with peritoneal dialysis scoring less than haemodialysis in social support. This is in line with the study conducted in Mexico by Chen et al. (2016), where they agreed that patients with good social support have a better health-related QoL, and are less frequently hospitalised than those with poor social support.

The low QoL observed in peritoneal dialysis patients is associated with a high frequency of dialysis, as they dialyse four to five times a day at home. For the haemodialysis patients, the low QoL is caused by the number of dialysis sessions, combined with the effects of travelling long distances for the dialysis sessions. Consistent with the study, patients have moral responsibilities to perform for their families, as the family unit is considered the cornerstone of a person’s well-being. Being unable to perform these roles disrupts the family unit both socially and economically (Senanayake et al. 2018). Providing support financially, emotionally, and socially when needed, plays a significant role in patients’ survival, as they feel that they are a burden to their families, mainly caused by financial dependence (Mbeje & Mtshali 2019).

The QoL of patients with ESRD is significantly compromised by financial constraints with 98.1% (*n* = 310) of patients being affected by economic factors such as poor personal and family income. Mbeje and Mtshali (2019) agreed that financial status affects the QoL of patients with ESRD, and all other aspects of their life. Mbeje and Mtshali (2019) further argued that loss of employment and financial dependence make patients anxious about their family’s survival, which results in a lack of confidence, leading to depression. Unemployment results in poor personal and family economic status, which is worsened by frequent visits to the hospital dialysis units, usually for a maximum of three times a week. The economic burden associated with ESRD has been researched in low-income and middle-income countries (LMICs), where it was found to be higher compared to developed countries. The rising burden of ESRD also increases the high cost of renal replacement therapy, which puts a strain on health systems globally (Kilonzo et al. 2017). Treatment expenses in LMICs are increased by late referrals when dialysis is already required, and are problematic when there is limited access to health care facilities that provide such services (George et al. 2017).

The findings in this study revealed that the general health of patients with ESRD affected aspects related to current health status, getting sick easily compared to others, feeling that the condition can become worse at any time, assuming that ESRD causes poor health, and comparing the current health status with 2 years ago. As these factors did not affect the QoL of patients with ESRD when comparing their current health status with 2 years ago, the general health domain scored high in the ESRD patients. Whether they are on peritoneal dialysis or haemodialysis, the patients’ physical state fluctuates as a result of the side-effects and physiological challenges that they encounter. This results in uncertainty about the future, thereby causing anxiety and depression at times, especially where there is poor social support (Ercan & Demir 2018).

The study conducted by Cruz et al. (2016) indicated that the QoL of patients in the early stages is much better than in the late stages of the disease, where they require dialysis to survive. In the current study, the patients were at the end stage of renal disease, and they all required renal replacement therapy (haemodialysis or peritoneal dialysis); hence, their QoL was significantly compromised. Responding to the patients’ needs, and dealing adequately with the factors affecting the QoL are essential to decreasing or delaying the mortality and morbidity rate as a result of ESRD and its complications.

### Limitations

The study was conducted in the public health institutions rendering nephrology services in a single province. The patients with ESRD undergoing dialysis in private institutions were not part of the study. The study has a limitation of information bias because the participants completed the questionnaires in the absence of the researcher. A face-to-face method of data collection might have helped in clarification of some information which the participants were not able to comprehend. Moreover, the use of unit managers to distribute and collect the questionnaires from participants might have contributed to information bias. Recall bias was quite possible, as the participants were asked to recall information that pertains to all the measured domains of QoL.

### Recommendations

The study findings indicate that there is a need to develop a framework to improve the QoL of patients with ESRD on dialysis. Strengthening collaboration between the health care providers and policymakers will enhance the implementation of strategies to improve the patients’ QoL. There is a need for further research on QoL to be conducted which will include the health care providers and families. Currently, doctors, nurses and families are the major role players in managing patients with ESRD. The government provides transport for patients from remote and rural areas to ease the financial burden; however, there is a need to explore the possibility of step-down units in decentralised sites and for centralised sites to be used for special and acute cases to reduce the patient load in these facilities.

## Conclusion

Although there is a high demand for renal replacement therapy in South Africa, the majority of patients with ESRD in the current study indicated a poor QoL. To address the physical, social, psychological and economic factors, and general health which have an impact on the patients’ QoL, health care personnel must be knowledgeable of the factors to intervene accordingly, using the available renal multidisciplinary health team. Furthermore, policymakers should initiate the decentralisation of renal services to other provincial hospitals, which will strengthen collaboration, knowledge sharing, building funding streams and budget to support each other.
